# Blood Adenosine Increase During Apnea in Spearfishermen Reinforces the Efficiency of the Cardiovascular Component of the Diving Reflex

**DOI:** 10.3389/fphys.2021.743154

**Published:** 2021-10-05

**Authors:** Marion Marlinge, Mohamed Chefrour, François Billaut, Marion Zavarro, Jean-Claude Rostain, Mathieu Coulange, Régis Guieu, Fabrice Joulia

**Affiliations:** ^1^C2VN, Center for Cardiovascular and Nutrition Research, INSERM 1263, INRAE 1260, Aix Marseille University, Marseille, France; ^2^Laboratory of Biochemistry, Timone Hospital, Marseille, France; ^3^Department of Kinesiology, Laval University, Quebec, QC, Canada; ^4^Faculty of Pharmacy, Marseille, France; ^5^Department of Hyperbaric Medicine, Hospital Sainte Marguerite, Marseille, France; ^6^UFR STAPS, Toulon University, La Garde, France

**Keywords:** adenosine, breath-hold, diving reflex, free-diving, hypoxia, training, spearfishing

## Abstract

The physiopathology consequences of hypoxia during breath-hold diving are a matter of debate. Adenosine (AD), an ATP derivative, is suspected to be implicated in the adaptive cardiovascular response to apnea, because of its vasodilating and bradycardic properties, two clinical manifestations observed during voluntary apnea. The aim of this study was to evaluate the adenosine response to apnea-induced hypoxia in trained spearfishermen (SFM) who are used to perform repetitive dives for 4–5 h. Twelve SFM (11 men and 1 woman, mean age 41 ± 3 years, apnea experience: 18 ± 9 years) and 10 control (CTL) subjects (age 44 ± 7 years) were enrolled in the study. Subjects were asked to main a dry static apnea and stopped it when they began the struggle phase (average duration: SFM 120 ± 78 s, CTL 78 ± 12 s). Capillary blood samples were collected at baseline and immediately after the apnea and analyzed for standard parameters and adenosine blood concentration ([AD]b). Heart rate (HR), systolic (SBP), and diastolic (DBP) blood pressures were also recorded continuously during the apnea. During the apnea, an increase in SBP and DBP and a decrease in HR were observed in both SFM and CTL. At baseline, [AD]b was higher in SFM compared with CTL (1.05 ± 0.2 vs. 0.73 ± 0.11 μM, *p* < 0.01). [AD]b increased significantly at the end of the apnea in both groups, but the increase was significantly greater in SFM than in controls (+90.4 vs. +12%, *p* < 0.01). Importantly, in SFM, we also observed significant correlations between [AD]b and HR (*R* = −0.8, *p* = 0.02), SpO_2_ (*R* = −0.69, *p* = 0.01), SBP (*R* = −0.89, *p* = 0.02), and DBP (*R* = −0.68, *p* = 0.03). Such associations were absent in CTL. The adenosine release during apnea was associated with blood O_2_ saturation and cardiovascular parameters in trained divers but not in controls. These data therefore suggest that adenosine may play a major role in the adaptive cardiovascular response to apnea and could reflect the level of training.

## Introduction

The cardiovascular adaptive response to breath-hold diving, also known as the diving response, has long been a matter of debate, especially since the popularization of recreational apnea and spearfishing. These aquatic activities commonly involve hours of immersion with repeated voluntary dynamic apneic phases causing severe hypoxia streak (Marlinge et al., 2021). The main cardiovascular responses occurring during breath-hold diving are bradycardia, peripheral vasoconstriction, and an increase in arterial blood pressure (Lindholm and Lundgren, 2009; Bain et al., 2018), while depending on the apnea duration, severe hypoxemia, and hypercapnia can also develop (Joulia et al., 2009). Several neurohumoral factors have also been suggested to be implicated in the cardiovascular response to hypoxemia, including cortisol, copeptin (Marlinge et al., 2019), catecholamines (Chmura et al., 2014; Eichhorn et al., 2017), and growth hormone (Djarova et al., 1986).

Adenosine (AD), an ATP derivative, is also implicated in the response to hypoxemia during breath hold (Joulia et al., 2013, 2014; Marlinge et al., 2019). Adenosine is released by endothelial cells and myocytes during hypoxia or ischemia. Indeed, with a drop in PaO_2_, the rephosphorylation of AD into ATP is limited because of the inhibiting action of the hypoxia-inducible factor (HIF) on adenosine kinases (Morote-Garcia et al., 2008). Adenosine thus accumulates in the extracellular spaces and strongly impacts the cardiovascular system through its four G-coupled membrane receptors named A_1_R, A_2*A*_R, A_2*B*_R, and A_3_R receptors. The activation of A_1_R can lead to bradycardia, sinus arrest, or atrioventricular block (ATVB), while the activation of A_2_ subtypes leads to strong vasodilation and hypotension. Moreover, A_3_R are implicated in ischemia–reperfusion protection (Guieu et al., 2020; Paganelli et al., 2021).

Interestingly, there are major differences between breath-hold divers (BHDs) and spearfishermen (SFM) in their average total apnea durations in 1 day of training or competition (BHD 39 ± 12 min vs. SFM 86 ± 39 min). Consequently, spearfishing training could be viewed as highly repetitive hypoxia exposure whereas shorter breath-hold training could be compared to acute hypoxia exposure, and there is a paucity of data on the responses of this population to hypoxia. Thus, the aim of this study was to evaluate the amplitude of the AD release and its role in the cardiovascular response to hypoxia. In SFM who are exposed to hypoxemia for several hours per week, we expected an accentuation of the adenosine release as well as an emphasis of the diving response compared to control (CTL).

## Materials and Methods

Twelve SFM (2 women and 10 men) with competitive experience at national and/or international levels and 10 CTL participants (1 woman and 9 men) volunteered to participate in this study (Table 1). All participants were non-smokers, without medical treatment and free from inflammatory or cardiovascular disease. The protocol was approved by our institutional Ethics Committee (CPP Sud Marseille N° 13/41) and the study was carried out by the Code of Ethics of the World Medical Association (in agreement with the Declaration of Helsinki). The procedures have been conducted with the adequate understanding and written consent of the participants.

**TABLE 1:** Spearfishermen (SFM, *n* = 12) and control (CTL, *n* = 10) characteristics.

	SFM	CTL
Age (years)	41 ± 3 (24–58)	44 ± 7 (35–55)
Men/women	10/2	8/2
Body mass (kg)	78 ± 11 (60–93)	78 ± 9 (57–90)
Height (cm)	177 ± 13 (162–190)	174 ± 12 (165–185)
Experience in spearfishing (years)	18 ± 9 (4–27)	0
Mean apnea duration per training session	86 ± 39 min	0
Training days per week	3 ± 2	0
Dry apnea duration (s)	120 ± 37 (90–220)	78 ± 9 (63–91)

*(Data are given as means, standard deviations, and range).*

### Protocol

The dry static apnea protocol has been described in detail elsewhere (Joulia et al., 2013, 2014). Briefly, all participants rested in supine position breathing normally in a controlled temperature room (25 ± 2°C) for 10 min. Apnea can be separated in two phases (Hentsch and Ulmer, 1984). The first one, or “*easy-going phase*” depends on physiological training adaptations whereas the second one known as the “*struggle phase*” is related to psychological capacity to fight against unpleasant feelings. To avoid the influence of training skills and allow better comparisons of physiological responses between SFM and CTL, participants were asked to perform a dry static apnea and to stop the breath hold when they began the struggle phase. The breath hold was also started without glossopharyngeal insufflation. The entire protocol was performed between 9 and 11 am.

Peripheral blood oxygen saturation (SpO_2_) and heart rate (HR) were measured continuously on the left index finger (NPB 40; Nellcor Puritan Bennett, Pleasanton, CA, United States). Systolic (SBP) and diastolic (DBP) blood pressures were measured before and at the end of the apnea session. Blood samples were collected on the right index at baseline and immediately after the apnea cessation after finger puncture for capillary analysis of pH, PCO_2_ and lactate concentration [La] (Epoc^®^, Blood Analysis System, Siemens). A separate drop of capillary blood was deposited on a blotting paper (Whatman^®^) for adenosine blood concentration ([AD]b) measurement as previously described (Marlinge et al., 2021).

### Statistical Analysis

Data’s distribution was tested using a Kolmogorov–Smirnov test. Since it did not reflect a normal distribution, the *U* Mann–Whitney test was used for inter-group comparisons (CTL vs. SFM) and the Wilcoxon matched-pair signed-Rank test for intra-group comparisons (before vs. after apnea). *P*-values < 0.05 were considered as significant. Analyses were performed with Statistica software 6.0.

## Results

There were no significant differences between SFM and CTL regarding their anthropometric characteristics (Table 1) and the resting values of HR, DBP, SBP, pH, SpO_2_, PCO_2_, and [La] (Table 2). As expected, the apnea durations were longer in SFM compared to CTL (Table 1).

**TABLE 2:** Biological and physical markers of spearfishermen (SFM) and control (CTL) recorded before and immediately at the end of the static apnea.

	SFM		CTL		
		
Biological markers	Baseline	Apnea	Baseline	Apnea	«b» *P*-values
Hb (g l^–1^)	15.4 ± 1.2	15.1 ± 0.8	15.4 ± 1.1	15 ± 1.3	NS
Hematocrit (%)	45 ± 1.7	46.4 ± 1.8	45.2 ± 1.4	44 ± 2	NS
Lactates (mM l^–1^)	1.10 ± 0.15	1.38 ± 0.14^*a,b*^	1.15 ± 0.3	1.16 ± 0.4	*P* < 0.01
SpO_2_ (mmHg)	110.5 ± 7^*b*^	87.2 ± 4.4^*a*^	96 ± 4	90 ± 4^*a*^	*P* < 0.01
Saturation (%)	99 ± 0.8	84 ± 7^*a,b*^	98 ± 0.4	90 ± 2^*a*^	*P* < 0.001
PCO_2_ (mmHg)	27.9 ± 2.5^*b*^	41 ± 2.9^*a*^	28 ± 1.8	37 ± 2 ^*a*^	*P* < 0.001
pH	7.41 ± 0.01	7.37 ± 0.01^*a*^	7.40 ± 0.02	7.37 ± 0.03^*a*^	*P* = 0.01
[AD]b (μM)	1.05 ± 0.2	2.1 ± 0.95^*a,b*^	0.59 ± 0.1	0.75 ± 0.1^*a*^	*P* = 0.01
**Cardiovascular parameters**					
SBP (mmHg)	116 ± 11.8	133 ± 14.4^*a*^	125 ± 6	131 ± 5^*a*^	*P* < 0.01
DBP (mmHg)	64 ± 8.9	71 ± 10.7^*a*^	70 ± 11	77 ± 6^*a*^	*P* < 0.01
HR (beats per min)	76 ± 3.9^*b*^	59 ± 6.2^*a,b*^	76 ± 5.2	67 ± 6.5	*P* < 0.01

*Data are given as mean ± SD. The symbol “a” was used when there is a significant difference between baseline and apnea values and “b” when there is a significant difference between SFM and CTL values.*

**FIGURE 1:**
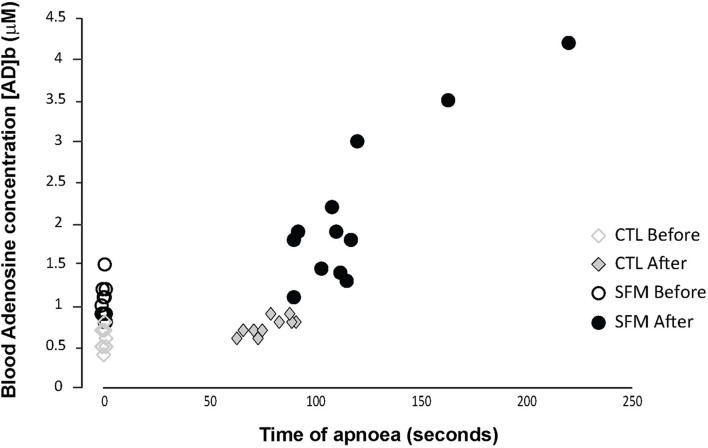
Individual values of blood adenosine concentration measured in spearfishermen (SFM) and control (CTL) subjects before and immediately at the end of the static apnea.

Individual apnea-induced changes in [AD]b are displayed in Figure 1. In basal conditions, [AD]b was higher in SFM compared with CTL (1.05 ± 0.2 vs. 0.61 ± 0.11 μM, *P* < 0.01). While [AD]b increased significantly at the end of apnea in both groups, the increase was greater in SFM compared to CTL (+1.05 vs. 0.16 μM, *P* < 0.01).

**FIGURE 2:**
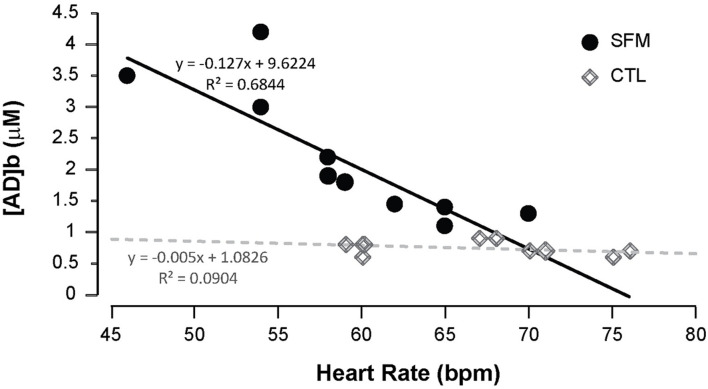
Correlations between the minimal heart rate recorded during the apnea and the blood adenosine concentration in spearfishermen (SFM) and control (CTL) subjects.

**FIGURE 3:**
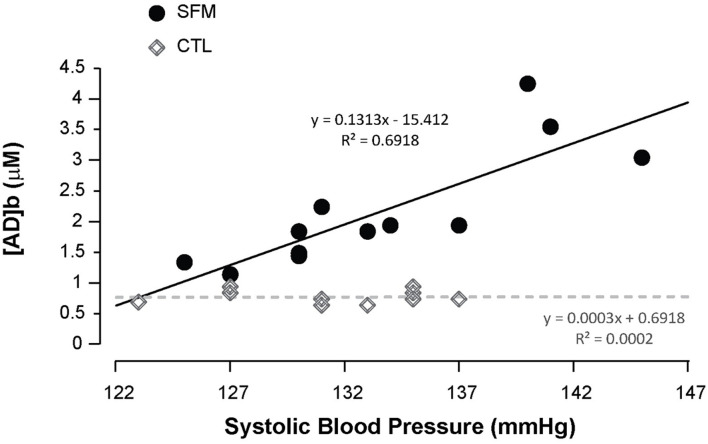
Correlations between the systolic blood pressure recorded during the apnea and the blood adenosine concentration in spearfishermen (SFM) and control (CTL) subjects.

**FIGURE 4:**
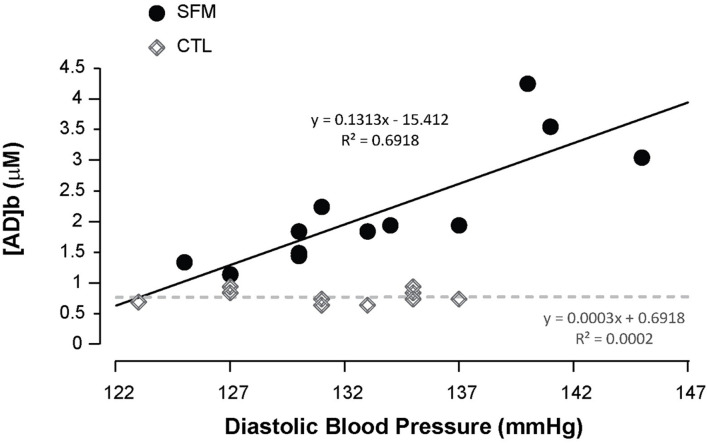
Correlations between the diastolic blood pressure recorded during the apnea and the blood adenosine concentration in spearfishermen (SFM) and control (CTL) subjects.

**FIGURE 5:**
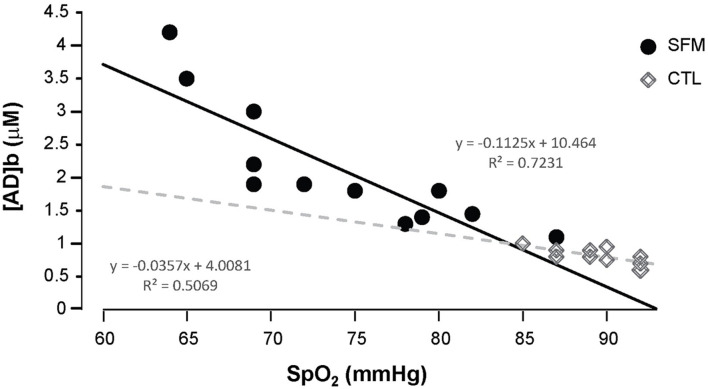
Correlations between the SpO_2_ values recording during the apnea and the blood adenosine concentration in spearfishermen (SFM) and control (CTL) subjects.

Apnea induced a bradycardia and increased in systolic (SBP) and diastolic (DBP) blood pressures in both SFM and CTL. The bradycardia was higher in SFM compared to CTL (−22 vs. −12%, *P* < 0.01) (Table 2). There was no significant difference between groups regarding DBP whereas the SBP increase was higher in SFM compared to CTL (17.1 vs. 6.2 mmHg, *P* < 0.01). Only in SFM, linear correlations were found between [AD]b measured at the end of the apnea and the cardiovascular parameters: HR (*R* = −0.83, *P* < 0.01, Figure 2), SBP (*R* = 0.83, *P* < 0.01, Figure 3), and DBP (*R* = 0.82, *P* < 0.01, Figure 4). Apnea induced a similar increase in PaCO_2_ in both SFM (+12.8 mmHg, *P* < 0.01) and CTL (+9 mmHg, *P* < 0.01). Apnea also induced a low but significant decrease of pH in both groups with no significant difference between groups (Table 2). Apnea generated a [La] increase in SFM only (+23%, *P* < 0.01). Although a decline in SpO_2_ was observed at the end of apnea in both groups, this decrease was significantly larger in SFM compared to CTL (−24 vs. −8.4%, *P* < 0.01). Finally, linear correlations were found between SpO_2_ and [AD]b in both SFM (*R* = −0.85, *P* < 0.01) and CTL (*R* = −0.71, *P* < 0.01, Figure 5).

## Discussion

This study investigated the response of blood AD to sub-maximal apnea in trained spearfishermen vs. novice control participants, and its association with key cardiovascular parameters. While the [AD]b increased in both groups during the apnea, SFM displayed a significantly greater increase. Importantly, this increase in [AD]b was correlated with the decline in HR as well as the increase in blood pressure in SFM only.

Such cardiovascular changes are well known to be part of the diving response (Ferrigno and Lundgren, 2010), which constitutes an integrative protective mechanism against hypoxia to decrease the work load on the heart and increase perfusion during the diastolic phase (Alboni et al., 2011). The diving reflex intensity is known to accentuate with the reduction in O_2_ saturation (Andersson and Schagatay, 1998; Andersson et al., 2002), the face immersion (Craig and Medd, 1968; Andersson et al., 2004), the depth (Ferrigno et al., 1991), and the level of expertise (Lemaître et al., 2010). Since participants only performed dry static apnea in our study, it could explain the significant but low bradycardia observed in SFM despite the apnea durations.

Vasoconstriction occurs during apnea in elite free-divers in order to limit skeletal muscle oxygen uptake and to facilitate blood redistribution toward the brain and the heart (Joulia et al., 2009). Vasoconstriction and the associated increase in systolic blood pressure are caused, at least in part, by the increase in epinephrine and norepinephrine concentrations during the apnea, and bradycardia is inversely correlated with the increase in norepinephrine (Eichhorn et al., 2017). Despite the bradycardia, the vasoconstriction is known to increase peripheric arterial pressure in well trained free-divers (Joulia et al., 2009). An [AD]b increase was previously described during apnea and higher in elite free-divers (Joulia et al., 2013), however, it is the first time that changes in [AD]b are correlated with the “intensity” of cardiovascular components of the diving reflex.

The high intensity of spearfishing training vs. free-diving training (Joulia et al., 2009) could induce a double protection against hypoxia during apneas. In fact, an accentuated diving reflex associated with a high [AD]b could limit the blood circulation in peripheral territories and subsequently increase heart and brain perfusion despite the cardiac output decrease and a skeletal muscles activation. Chronic hypoxia exposure increases circulating AD in the blood which acts on A_1_R situated on proximal and terminal skeletal muscle arterioles (Marshall, 2001), thereby limiting the blood pressure increase. The maintenance of the peripheral vasoconstrictor reflex during apnea in SFM, despite the high increase in [AD]b, is favoring the increase in brain perfusion previously describe in free-divers (Joulia et al., 2009; Vestergaard and Larsson, 2019). The [AD]b increase on A_2_R, the predominant receptor subtype responsible for coronary blood flow regulation, induces a coronary arteries dilatation favoring the myocardial perfusion whereas its effect on the A_1_R in supraventricular tissues (atrial myocytes, sinoatrial node, and atrioventricular node) exerts a negative chronotropic effect by suppressing the automaticity of cardiac pacemakers, and a negative dromotropic effect through inhibition of AV-nodal conduction (Mustafa et al., 2009). Finally, since the arterial blood pressure increase and the bradycardia were moderate in CTL and not correlated with the slight increase in [AD]b, we can hypothesize that the brain and heart in this population would be less protected against apnea-induced hypoxia even during short apnea durations.

The [AD]b increase was found to be secondary to the decrease in the erythrocyte nucleoside transporter 1 (ENT1) that regulates the extracellular concentration of AD, and which is down-regulated in free-divers thereby preventing AD uptake by erythrocytes and leading to an increase in [AD]b (Marlinge et al., 2021). It is likely that [AD]b is not simply correlated with HR and blood pressure, but actively participates in the control of the cardiovascular system since it is well established that its endogenous increase, *via* the activation of A_1_R, decreases heart rhythm and causes vasodilation through A_2_A or A_2_B receptor activation (Saadjian et al., 2009; Guieu et al., 2020; Paganelli et al., 2021). Furthermore, exogenous administration of AD leads to bradycardia, sinus arrest, and sometimes ATVB (Brignole et al., 2003; Guieu et al., 2015; Deharo et al., 2018). Interestingly, the increase in [AD]b in control participants does not seem sufficient to induce vasodilation. Indeed, the *K*_*D*_ for the activation of A_2*A*_R, the main receptors implicated in vasodilation, is in the range of 1.8 μM (Shryock et al., 1998). Such a concentration is usually reached only during prolonged apnea (Marlinge et al., 2021) which is only the case for the SFM group in our study.

Conversely, the [AD]b necessary to activate A_1_R is in the range of 0.8 μM (Cohen et al., 1996). This may explain the bradycardia observed both in SFM and CTL at the end of the apnea in the current study. In basal condition, however, the relatively high [AD]b measured in divers was not correlated with HR and there was no difference between basal HR and arterial blood pressure recorded in SFM and CTL. This is suggesting that high basal [AD]b could be associated with a down regulation of AD receptors as previously reported in participants suffering from vasovagal manifestations and chronically exposed to high basal [AD]b (Brignole et al., 2017) and/or a compensating increase in sympathetic activity and catecholamine release to counterbalance the bradycardic and peripheric vasodilator effects of [AD]b (Dibner-Dunlap et al., 1993). Finally, since it was recently shown that the succession of oxygen partial pressure variations as hyperoxia–normoxia succession could modify the regulation of the HIF transcription factor activity (Fratantonio et al., 2021) we can suppose that the succession of apneas during spearfishing could modify the HIF transcription activity known to be linked to the rephosphorylation of AD into ATP (Morote-Garcia et al., 2008).

In conclusion, the AD release during apnea seems to play a major role in the adaptive cardiovascular response to hypoxia. The release of AD in the bloodstream, triggered by hypoxia, limits the increase in blood pressure and maintains sufficient flow to key organs through its vasodilation properties, and further protects the myocardium *via* its HR slowing effects. Finally, the AD release observed in SFM was higher compared to the one observed in free-divers suggesting that spearfishing training is more efficient to increase the diving response than apnea training.

## Data Availability Statement

The raw data supporting the conclusions of this article will be made available by the authors, without undue reservation.

## Ethics Statement

The studies involving human participants were reviewed and approved by the CPP Sud Marseille N° 13/41. The patients/participants provided their written informed consent to participate in this study.

## Author Contributions

FB, RG, FJ, and MM conceived and designed the study. MCh and MZ performed the molecular biology and recruited the participants. FB, MCo, RG, FJ, and J-CR critically revised the manuscript. All authors contributed to the article and approved the submitted version.

## Conflict of Interest

The authors declare that the research was conducted in the absence of any commercial or financial relationships that could be construed as a potential conflict of interest.

## Publisher’s Note

All claims expressed in this article are solely those of the authors and do not necessarily represent those of their affiliated organizations, or those of the publisher, the editors and the reviewers. Any product that may be evaluated in this article, or claim that may be made by its manufacturer, is not guaranteed or endorsed by the publisher.
